# Supplementary material for: Retinal Pigment Epithelial Cells Derived from Induced Pluripotent Stem (iPS) Cells Suppress or Activate T Cells via Costimulatory Signals
Source: Int J Mol Sci. 2020 Sep 5;21(18):6507. doi: 10.3390/ijms21186507 (PMC7554762; doi:10.3390/ijms21186507)
Supplement: Supplementary file 1 [file ijms-21-06507-s001.pdf]

Supplementary materials

Fig. S1.

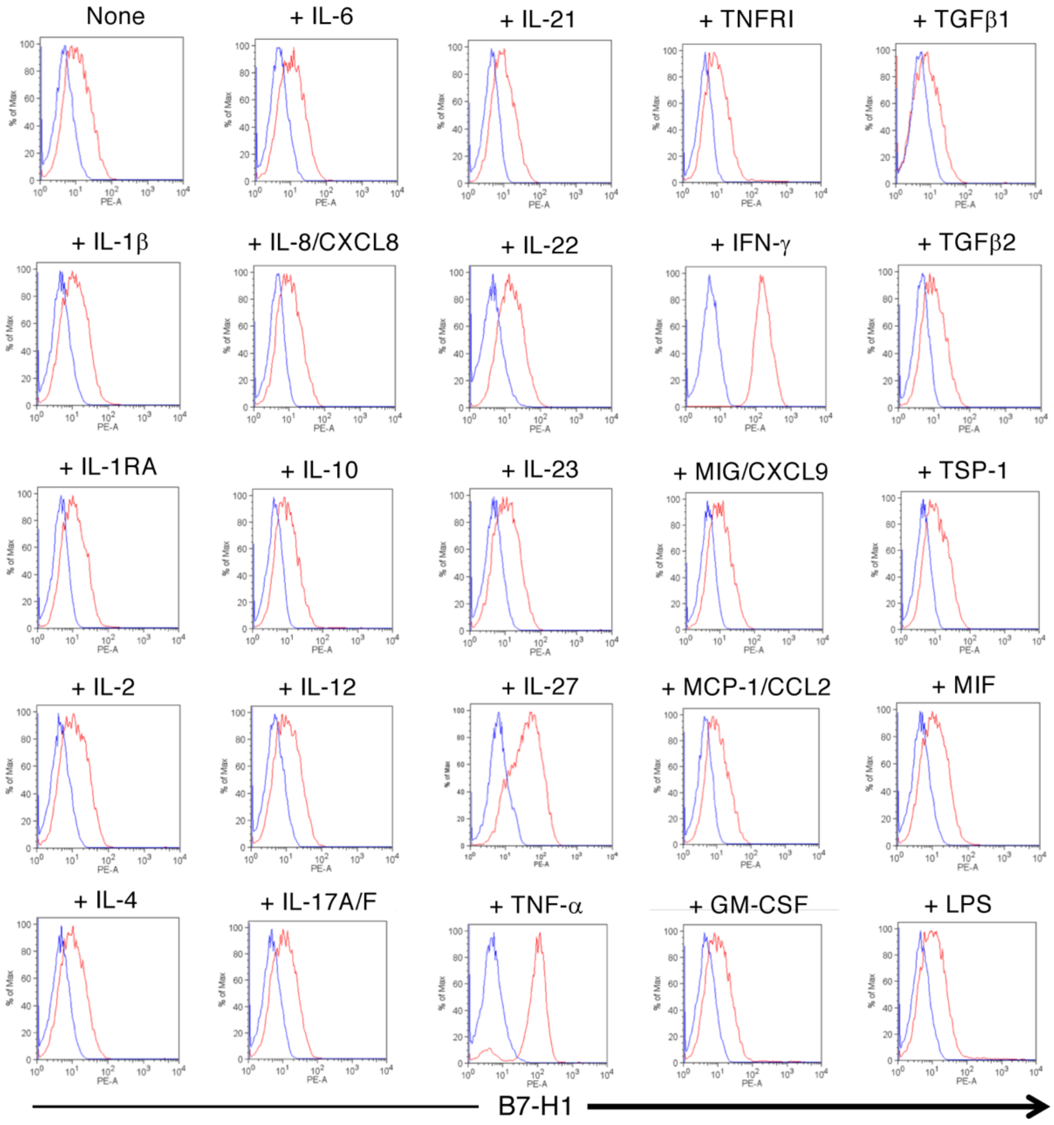

Fig. S1. Expression of B7-H1/CD274 on human iPS-RPE cells in the presence of inflammatory proteins.

To confirm the expression of B7-H1/CD274 on human iPS-RPE cells, human recombinant proteins such as IL-1β, IL-1RA, IL-2, IL-4, IL-6, IL-8, IL-10, IL-12, IL-17A/F, IL-21, IL-22, IL-23, IL-27, TNF-α, TNFR1, IFN-γ, MIG, MCP-1, GM-SCF, TGFβ1, TGFβ2, TSP-1, MIF, and LPS were used prior to FACS analysis. As a result, iPS-RPE cells exposed to IFN-γ, TNF-α, and IL-27 had inducibly expressed B7-H1. Blue histogram: data for isotype control. TSP-1: thrombospondin-1; MI: macrophage migration inhibitory factor; LPS: lipopolysaccharide.

Fig. S2.

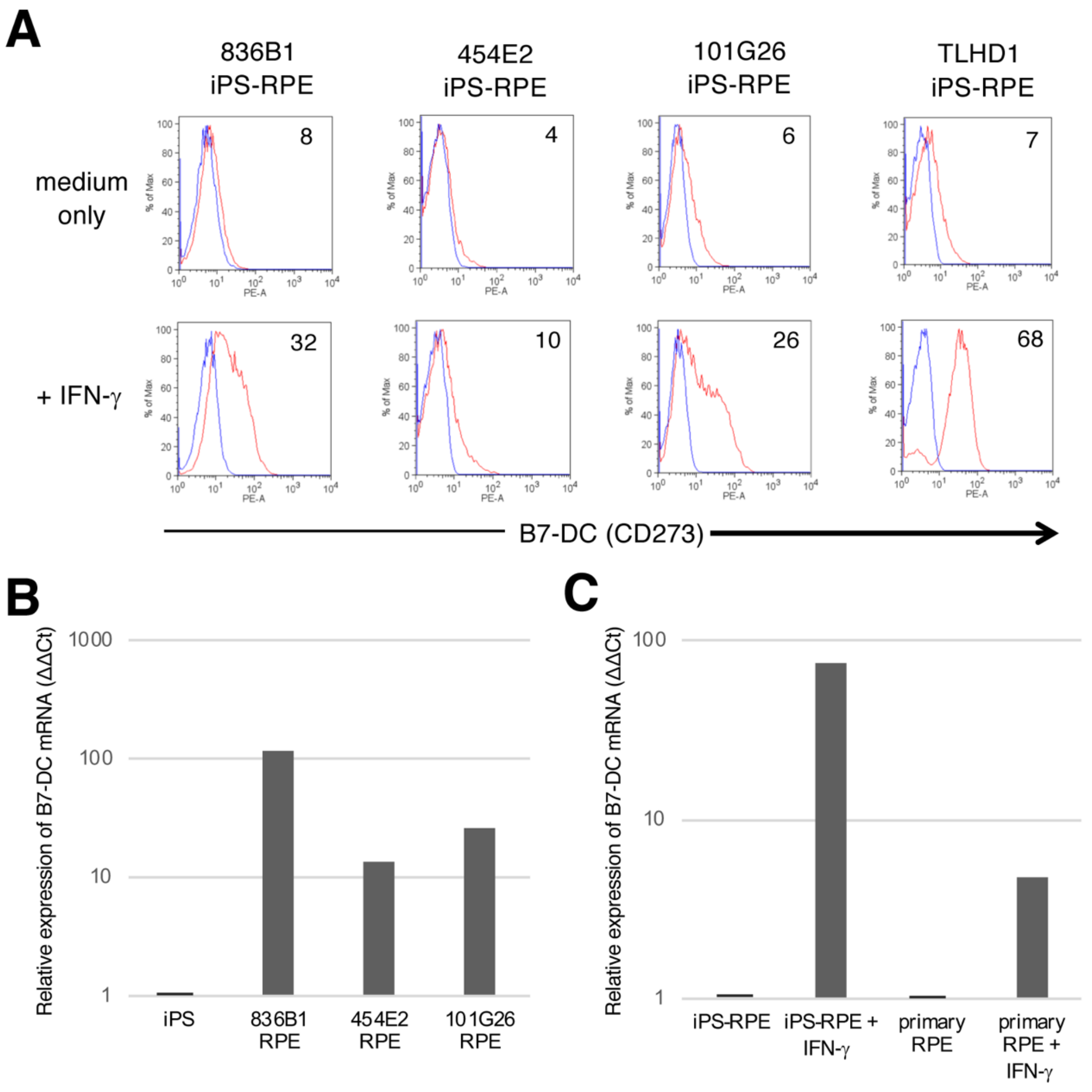

Fig. S2. Expression of B7-DC/CD273 on human iPS-RPE cells.

We examined whether iPS-RPE cells expressed B7-DC. (**A**) In FACS results, iPS-RPE cells poorly expressed B7-DC. However, IFN-γ-pretreated iPS-RPE cells clearly expressed it. The numbers in the histograms indicate MFI. Blue histogram: data for isotype control. (**B**) In qRT-PCR, iPS-RPE cells expressed mRNA for B7-DC compared with control iPS cells. (**C**) In addition, IFN-γ-pretreated iPS-RPE cells, as well as primary RPE cells, highly expressed the molecules compared with non-treated RPE cells. These results suggest that RPE cells express the co-stimulatory molecules, especially under inflammatory conditions.

Fig. S3.

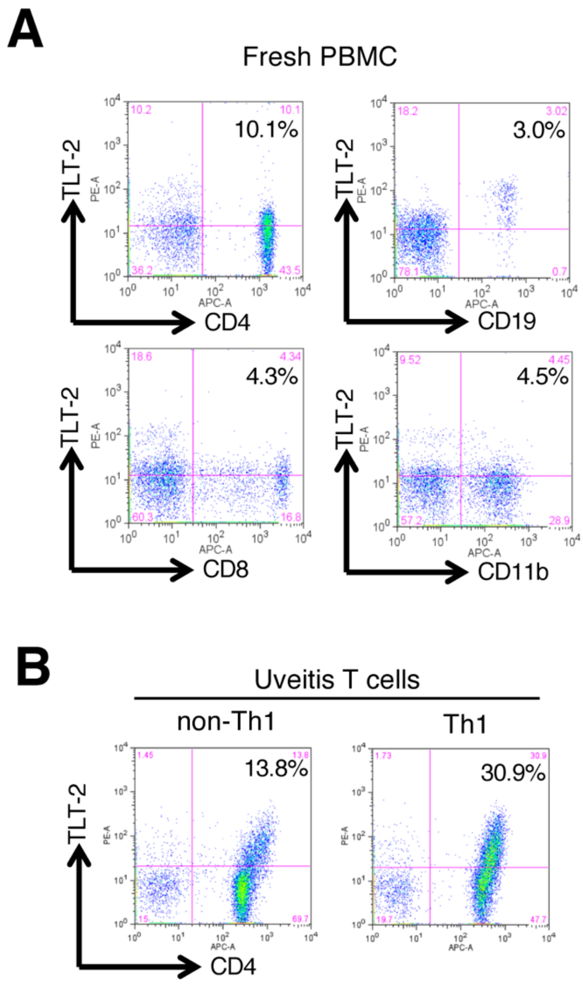

Fig. S3. Expression of TLT-2 on T cells.

(**A**) FACS showed that T cells (CD4 and CD8), monocytes/macrophages (CD11b), and B cells (CD19) in PBMC from a healthy donor expressed TLT-2 receptor, especially CD4$^+$ T cells. (**B**) In addition, non-Th1-type CD4$^+$ T cells from a VKH uveitis patient expressed TLT-2. However, Th1-type CD4$^+$ T cells from the patient highly expressed TLT-2 on their surfaces. Numbers in the histograms indicate the percentage of cells double-positive (e.g., CD4 and TLT-2). TLT-2: triggering receptor expressed on myeloid cell-like transcript 2.

Fig. S4.

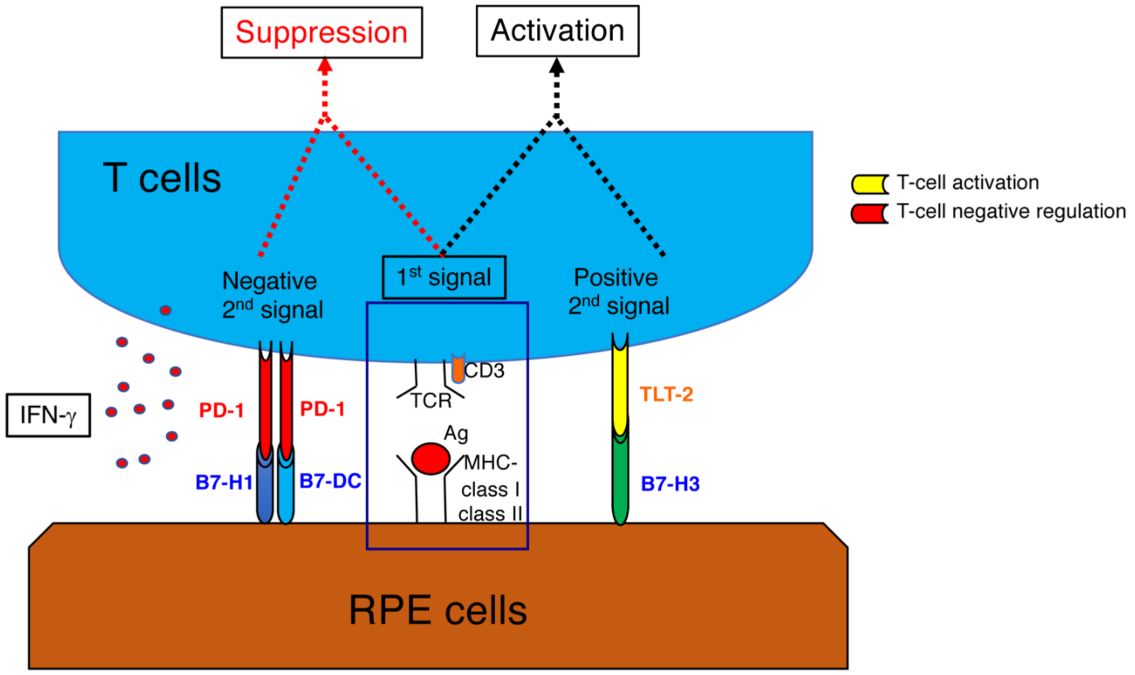

Fig. S4. Summary of the relationship between RPE cells and T cells with 2nd co-stimulatory signals.

The first signal is the interaction of the T-cell receptor (TCR) with antigen (Ag) peptide-MHC complexes presented by RPE cells. B7-H3 co-stimulatory molecules on RPE cells are able to activate/stimulate T cells through receptors such as TLT-2. By contrast, B7-H1/B7-DC negative co-stimulatory molecules on RPE cells are able to suppress bystander T cells through the PD-1 receptor. IFN-γ cytokines secreted by T cells, especially Th1 cells, are necessary for the interaction. The first signal (MHC-TCR complex) is require for T-cell regulation together with these 2nd co-stimulatory signals.

Table S1. Antibody information

No.	Antibody	Species	Concentration	Application / Tested cells	Source
1	anti-human CD3	Mouse	1 μg/mL	*In vitro* assay / T cells	Ancell
2	anti-human CD4, APC	Mouse	2 μl/test	FACS / T cells, PBMC	Miltenyi Biotec
3	anti-human CD8a, APC	Mouse	5 μl/test	FACS / T cells, PBMC	eBioscience
4	anti-human CD11b, APC	Rat	2 μl/test	FACS / monocytes, PBMC	Miltenyi Biotec
5	anti-human CD19, APC	Mouse	10 μl/test	FACS / B cells, PBMC	BD PharMingen
6	anti-human IFN-γ, PE	Mouse	10 μl/test	FACS / T cells	R&D systems
7	anti-human CD40, PE	Mouse	5 μl/test	FACS / RPE ells	BioLegend
8	anti-human ICAM-1/CD54, FITC	Mouse	10 μl/test	FACS / RPE ells	R&D systems
9	anti-human CD70 (CD27-L), PE	Mouse	5 μl/test	FACS / RPE ells	BioLegend
10	anti-human CD80 (B7-1), FITC	Mouse	5 μl/test	FACS / RPE ells	eBioscience
11	anti-human CD86 (B7-2), FITC	Mouse	5 μl/test	FACS / RPE ells	eBioscience
12	anti-human CD252 (OX40L), PE	Mouse	5 μl/test	FACS / RPE ells	BioLegend
13	anti-human CD270 (HVEM), PE	Mouse	5 μl/test	FACS / RPE ells	BioLegend
14	anti-human CD273 (B7-DC), PE	Mouse	5 μl/test	FACS / RPE ells	eBioscience
15	anti-human CD274 (B7-H1), PE	Mouse	5 μl/test	FACS / RPE ells	eBioscience
16	anti-human CD274 (B7-H1), purified	Rat	×100	Immunohistochemistry / RPE	eBioscience
17	anti-human CD275 (B7-H2), PE	Mouse	5 μl/test	FACS / RPE cells	eBioscience
18	anti-human CD276 (B7-H3), PE	Mouse	5 μl/test	FACS / RPE cells	BioLegend
19	anti-human CD276 (B7-H3), purified	Mouse	1 μg/ml	*In vitro* assay	BioLegend
20	anti-human CD276 (B7-H3), purified	Goat	×100	Immunohistochemistry / RPE	R&D systems
21	anti-human B7-H4, PE	Mouse	5 μl/test	FACS / RPE cells	eBioscience

22	anti-human Fas ligand (TNFSF6), PE	Mouse	5 µl/test	FACS / RPE cells	BioLegend
23	anti-human 4-1BB-L (TNFSF9), PE	Mouse	10 µl/test	FACS / RPE cells	R&D systems
24	anti-human GITRL (TNFSF18), PE	Mouse	10 µl/test	FACS / RPE cells	R&D systems
25	anti-human PD-1 (CD279), PE	Mouse	10 µl/test	FACS / T cells, PBMC	BD PharMingen
26	anti-human PD-1 (CD279), purified	Mouse	1 µg/ml	*In vitro* assay	BD PharMingen
27	anti-human TLT-2, PE	Mouse	5 µl/test	FACS / T cells, PBMC	BioLegend
28	anti-human Ki-67, PE	Mouse	5 µl/test	FACS /T cells, PBMC	BioLegend
29	anti-rat IgG, Alexa Fluor 546	Goat	×1000	Immunohistochemistry (2nd abs) / RPE	Invitrogen
30	anti-goat IgG, Alexa Fluor 488	Donkey	×1000	Immunohistochemistry (2nd abs) / RPE	Invitrogen
31	4′,6-diamidino-2-phenylindole (DAPI)	-	×1000	Immunohistochemistry (counterstaining)	Invitrogen
32	mouse IgG1κ, purified	-	1 µg/ml	*In vitro* assay (isotype control)	BD PharMingen
33	mouse IgG1κ, purified	-	1 µg/ml	*In vitro* assay (isotype control)	BioLegend
34	mouse IgG, PE	-	5 µl/test	FACS / T cells, PBMC	BioLegend
35	mouse IgG, PE	-	5 µl/test	FACS / RPE cells	BioLegend
36	mouse IgG, PE	-	5 µl/test	FACS / RPE cells	eBioscience
37	mouse IgG, FITC	-	10 µl/test	FACS / RPE ells	R&D systems
38	mouse IgG, PE	-	10 µl/test	FACS / T cells	R&D systems
39	mouse IgG, APC	-	2 µl/test	FACS / T cells, PBMC	Miltenyi Biotec
40	mouse IgG, APC	-	10 µl/test	FACS / B cells, PBMC	BD PharMingen
41	anti-human IL-4	Rat	5 µg/mL	*In vitro* assay / T cells	eBioscience

Table S2. Primers and probes for qRT-PCR

No.	Molecule	Left primer	Right primer	Probe*
1	actin, beta (β-actin)	ccaaccgcgagaagatga	ccagaggcgtacagggatag	#64
2	programmed cell death 1 ligand 1 (PD-L1/B7-H1/CD274)	ccatacagctgaattggtcatc	cagaattaccaagtgagtcctttca	#88
3	programmed cell death 1 (PDCD1: PD-1)	ctccaggcatgcagatcc	ggcctgtctggggagtcta	#26
4	CD276 molecule (B7-H3)	tgggagcactgtggttctg	agtgccaccactgggtctt	#85
5	programmed cell death 1 ligand 2 (PD-L2/B7-DC/CD273)	gagctgtggcaagtcctcat	gcaattccaggctcaacatta	#69

*Probe - The probe in the Roche Universal Probe Library was used for qRT-PCR assay.

Table S3. Recombinant human proteins

No.	Recombinant protein	Concentration	Application / Tested cells	Source
1	recombinant human IL-2	100 U/ml	*In vitro* assay / T cells, PBMC, RPE cells	BD Biosciences
2	recombinant human IL-1β	50 ng/ml	*In vitro* assay / RPE cells	R&D systems
3	recombinant human IL-1RA	50 ng/ml	*In vitro* assay / RPE cells	R&D systems
4	recombinant human IL-4	50 ng/ml	*In vitro* assay / RPE cells	BD PharMingen
5	recombinant human IL-6	50 ng/ml	*In vitro* assay / RPE cells	R&D systems
6	recombinant human IL-8/CXCL8	50 ng/ml	*In vitro* assay / RPE cells	R&D systems
7	recombinant human IL-10	50 ng/ml	*In vitro* assay / RPE cells	R&D systems
8	recombinant human IL-12	50 ng/ml	*In vitro* assay / RPE cells, T cells	R&D systems
9	recombinant human IL-17A/F	50 ng/ml	*In vitro* assay / RPE cells	R&D systems
10	recombinant human IL-21	50 ng/ml	*In vitro* assay / RPE cells	eBioscience
11	recombinant human IL-22	50 ng/ml	*In vitro* assay / RPE cells	R&D systems
12	recombinant human IL-27	50 ng/ml	*In vitro* assay / RPE cells	R&D systems
13	recombinant human TNF-α	50 ng/ml	*In vitro* assay / RPE cells	R&D systems
14	recombinant human TNFR1	50 ng/ml	*In vitro* assay / RPE cells	R&D systems
15	recombinant human IFN-γ	0.1, 1, 10, 100 ng/ml	*In vitro* assay / RPE cells, T cells	R&D systems
16	recombinant human MIG/CXCL9	50 ng/ml	*In vitro* assay / RPE cells	R&D systems
17	recombinant human MCP-1/CCL2	50 ng/ml	*In vitro* assay / RPE cells	R&D systems
18	recombinant human GM-CSF	50 ng/ml	*In vitro* assay / RPE cells	BD PharMingen
19	recombinant human TGFβ1	50 ng/ml	*In vitro* assay / RPE cells	R&D systems
20	recombinant human TGFβ2	50 ng/ml	*In vitro* assay / RPE cells	R&D systems

21	recombinant human TSP-1	50 ng/ml	*In vitro* assay / RPE cells	R&D systems
22	recombinant human MIF	50 ng/ml	*In vitro* assay / RPE cells	R&D systems
23	lipopolysaccharide (LPS)	50 ng/ml	*In vitro* assay / RPE cells	Sigma-Aldrich
24	recombinant human B7-H3/CD276	0.5, 5, 20, 200 ng/ml	*In vitro* assay / T cells, PBMC	R&D systems

TSP-1: thrombospondin-1; MIF: macrophage migration inhibitory factor.
